# Phenylethyl Alcohol-Based Polymeric Nanogels Obtained Through Polymerization-Induced Self-Assembly Toward Achieving Broad-Spectrum Antibacterial Activity

**DOI:** 10.3390/gels11090690

**Published:** 2025-09-01

**Authors:** Rui Xie, Xinru Gao, Ketao Liu, Deshui Yu, Qiaoran Li, Guang Yang, Feihu Bi

**Affiliations:** 1 Biomass Molecular Engineering Center, Department of Materials Science and Engineering, Anhui Agricultural University, Hefei 230036, China; 21710013@stu.ahau.edu.cn (R.X.); gaoxinru129@stu.ahau.edu.cn (X.G.); 17681063345@163.com (K.L.); deshuiyu@yeah.net (D.Y.); guangyang@ahau.edu.cn (G.Y.); 2 School of Life Sciences, Jinggangshan University, Ji’an 343009, China

**Keywords:** polymerization-induced self-assembly, polymer nanogels, antibacterial, abiotic resistance

## Abstract

The emergence of bacterial resistance has spurred an urgent need to develop effective alternatives to traditional antibiotics. Phenylethyl alcohol from plants exhibits potential antimicrobial properties, but its efficacy is limited due to its compromised dispersion in water and structural stability in ambient conditions. Herein, for the first time, a polymerization-induced self-assembly strategy was employed to obtain different morphological nanogels with phenylethyl alcohol moieties as hydrophobic cores through in situ reversible addition–fragmentation chain-transfer (RAFT) polymerization. The well-defined copolymers of PTEG_x_-*co*-PPMA_y_ with controllable molecular weights and narrow polydispersity were confirmed by a combination of techniques. The generated phenylethyl alcohol-based nanogels demonstrated potent antibacterial activity, particularly PTEG_30_-*co*-PPMA_70_ with a one-dimensional linear architecture, which achieved a minimum inhibitory concentration of 62 μg mL^−1^ against *E. coli*. SEM revealed membrane disruption as the bactericidal mechanism, highlighting enhanced efficacy against Gram-negative bacteria due to structural differences in cell envelopes. This study establishes a robust platform for designing phenylethyl alcohol-based nanogels with controllable structures toward achieving potent antimicrobial performance, offering a promising strategy for combating bacterial resistance while addressing the dilemma of conventional antibiotic drug systems.

## 1. Introduction

Bacterial pathogens remain a critical global health concern due to their capacity to induce severe infections characterized by pyrexia, inflammatory responses, and systemic discomfort [1,2]. Conventional therapeutic approaches employing empirical antibiotic regimens have inadvertently accelerated the emergence of multidrug-resistant microbial strains through evolutionary selection pressure [3]. Contemporary studies have explored advanced biocidal materials, including polymeric quaternary ammonium compounds [4,5] and engineered covalent organic frameworks [6,7], as potential antimicrobial alternatives [8]. Nevertheless, these synthetic agents raise ecological and clinical concerns regarding their non-biodegradable nature and potential cytotoxic effects from metabolic byproducts in biological systems [9,10]. This urgent clinical challenge necessitates the development of innovative therapeutic strategies incorporating biocompatible, ecologically sustainable antimicrobial agents with minimized off-target toxicity.

Phenylethyl alcohol (PA), a plant-based molecule predominantly located in floral species like roses [11], exhibits potential bactericidal activity, which stems from membrane disruption mechanisms, where it permeates microbial cell barriers and accelerates potassium ion efflux [12]. In vivo studies further confirm its antioxidative potential [13,14]. Beyond antimicrobial applications, PA shows promise as a fumigation agent for inhibiting fungal contamination in edible seed preservation. However, its practical utilization faces challenges due to its poor water dispersion capacity and chemical instability, necessitating advanced formulation strategies. Recent research has explored nanoliposomal (NLP) encapsulation as a delivery enhancement approach [15,16], building on previous successes with liposomal systems in optimizing dermal permeation and pharmacological efficacy. Such vesicular carriers have proven particularly valuable in wound care therapeutics, where they protect bioactive agents while facilitating targeted release [17]. In previous studies, phenethyl alcohol was loaded into NLPs using the thin-film hydration method, achieving an encapsulation efficiency of up to 90%, which enhanced its aqueous solubility and utilization efficiency [18]. However, the NLP encapsulation strategy may be associated with the issues of rapid release and the low release rate of phenethyl alcohol.

Polymer nanogels constitute three-dimensional nanoscale networks formed through physical or chemical cross-linking of polymer chains [19]. Recognized as promising drug delivery systems, these nanostructures offer advantageous properties, including biocompatibility, high stability, tunable particle dimensions, and facile surface functionalization for targeted release [20]. Such networks may be stabilized by either covalent bonds between polymer chains or non-covalent interactions [21,22]. Polymerization-induced self-assembly (PISA) represents a powerful technique for the direct synthesis of high-concentration soft nanogels with precisely tunable dimensions and architectures, offering industrial scalability [23,24,25]. This process primarily relies on hydrophobic forces to drive the autonomous organization of block copolymers (BCPs), with the resulting nanogels’ evolution being governed by the thermodynamic hydration parameters of the hydrophobic segments, which correlate with their degree of polymerization [26,27]. Recent advances have established PISA as a robust platform for fabricating therapeutic nanovehicles featuring tailorable and industrially feasible (≤50% solids) characteristics through controlled BCP synthesis. Structural polymorphism across spherical micelles, elongated worms, and hollow vesicles can be achieved by manipulating the amphiphilicity balance of the polymeric components, demonstrating reversible morphological shifts [28]. While these PISA-derived nanogels show significant potential in biomedical cargo transport, they face limitations. Drug loading within these nanogels is primarily achieved through passive permeation or encapsulation, which often results in suboptimal drug payloads and challenges in achieving spatiotemporal control over drug release. These constraints ultimately pose substantial challenges to nanogel design [29,30]. Addressing these constraints, innovative approaches propose using pharmacologically active monomers as building blocks for carrier synthesis [31], enabling inherent therapeutic payload integration and maximized utilization efficiency [32].

**Scheme 1:**
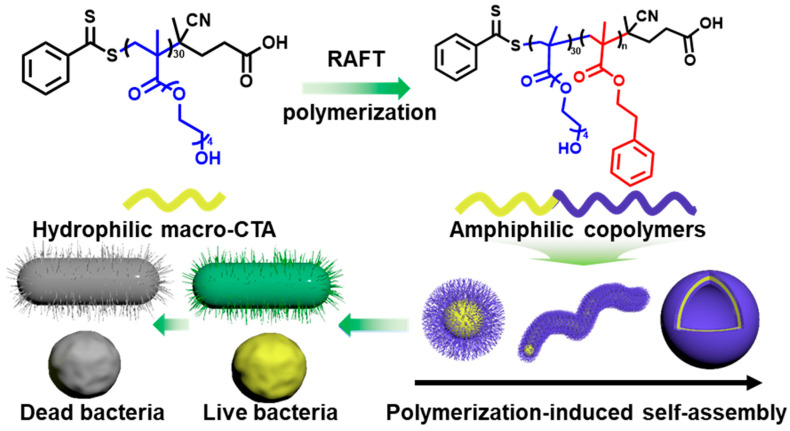
Illustration of fabrication procedures of phenylethyl alcohol-based polymeric nanogels and removal of bacteria.

This study developed a bioactive monomer strategy for the direct integration of a pharmacophore-derived monomer from PA into an amphiphilic block copolymer (PTEG_30_-*co*-PPMA_n_) via RAFT polymerization. Leveraging PISA, precise morphological control was achieved, yielding nanogels ranging from spherical micelles (33 nm) to vesicles (326 nm), while imparting inherent antibacterial functionality. The optimized PTEG_30_-*co*-PPMA_70_ copolymer exhibited broad-spectrum bactericidal activity by disrupting cell membranes (Scheme 1). This validates the therapeutic monomer approach as a versatile platform for developing integrated antibacterial nanocarriers with dual carrier–bioactive functions.

## 2. Results and Discussion

### 2.1. Synthesis and Characterization of Phenylethyl Alcohol-Based Polymers

**Figure 1:**
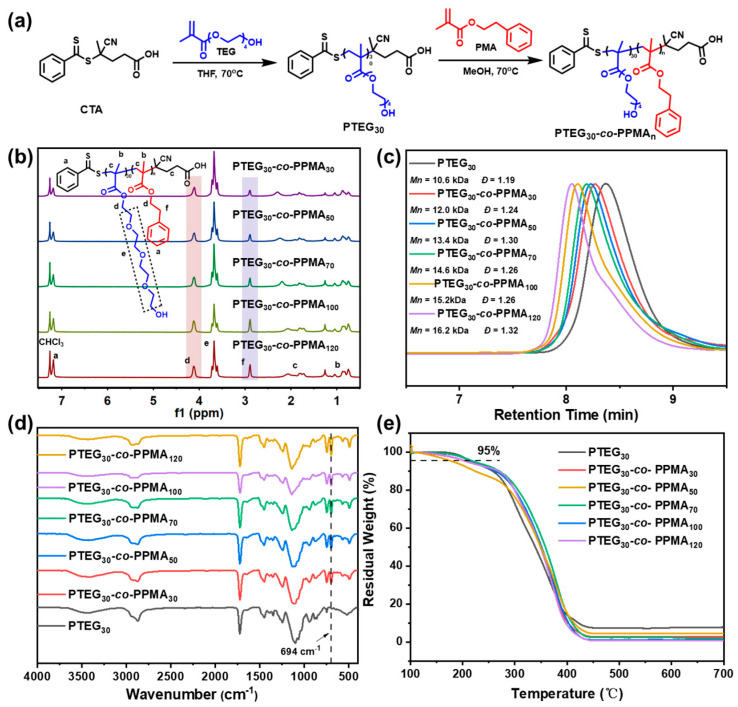
Synthesis and characterization of amphiphilic block copolymer PTEG_30_-PPMA_n_. (**a**) Synthetic routes of PTEG_30_-*co*-PPMA_n_. (**b**) ^1^H NMR spectra of PTEG_30_-*co*-PPMA_n_. (**c**) GPC curves of PTEG_30_-*co*-PPMA_n_. (**d**) FT-IR curves of PTEG_30_ and PTEG_30_-*co*-PPMA_n_. (**e**) TGA curves of PTEG_30_ and PTEG_30_-*co*-PPMA_n_.

The amphiphilic copolymers PTEG_30_-*co*-PPMA_n_, characterized by varying molecular weights and controllable molecular structures, were synthesized using reversible addition−fragmentation chain-transfer (RAFT) polymerization, as illustrated in Figure 1a. Initially, the RAFT polymerization of TEG was employed to create a hydrophilic macromolecular initiator, which played a crucial role in the subsequent polymerization process. The chemical structure of PTEG_30_ was confirmed by ^1^H NMR spectroscopy. Based on the integral ratio of the signals at 7.85 ppm and 4.2 ppm, the conversion of the TEG monomer was determined to be 93.3%, yielding an actual degree of polymerization (DP) of 28. The relevant results are shown in Figure S1. Gel permeation chromatography (GPC) analysis revealed that the molecular weight (*M*_n_) of PTEG_30_ was 10.6 kDa, accompanied by a polydispersity index (*M*_w_/*M*_n_, *Đ*) of 1.19, as detailed in Figure 1c.

To synthesize block copolymers (PTEG_30_-*co*-PPMA_n_) with varying molecular weights, a series of parallel experiments was conducted, each utilizing different molar ratios of PTEG_30_ and PMA. The molecular structures and weights of the resulting copolymers were determined through a combination of advanced characterization techniques. ^1^H NMR microscopy was employed to elucidate the structure of PTEG_30_-*co*-PPMA_n_, as shown in Figure 1b. The ^1^H NMR spectra exhibited distinct signals corresponding to the PPMA and PTEG_30_ block segments, the signal at 3.0–2.8 ppm corresponded to the benzylic methylene protons (-CH_2_- adjacent to the phenyl ring) in PTEG_30_-*co*-PPMA_n_, and the signal at 4.2–4.0 ppm was attributed to the methylene protons adjacent to the ester linkage (-CH_2_- adjacent to the ester group) in PTEG_30_-*co*-PPMA_n_. Based on the integral ratio of these two signals, the actual DP of PTEG_30_-*co*-PPMA_n_ was determined. The conversion of the PMA monomer was found to be between 51.4% and 66.7%, indicating the successful synthesis of glycoblock copolymers (Table S1). The GPC traces further confirmed a narrow molecular weight distribution for each polymer, as depicted in Figure 1c. Specifically, the molecular weights and polydispersity indices for the various copolymers were as follows: PTEG_30_-*co*-PPMA_30_ (*M*_n_ = 12.0 kDa; *Đ* = 1.24), PTEG_30_-*co*-PPMA_50_ (*M*_n_ = 13.4 kDa; *Đ* = 1.30), PTEG_30_-*co*-PPMA_70_ (*M*_n_ = 14.6 kDa; *Đ* = 1.26), PTEG_30_-*co*-PPMA_100_ (*M*_n_ = 15.2 kDa; *Đ* = 1.26), and PTEG_30_-*co*-PPMA_120_ (*M*_n_ = 16.2 kDa; *Đ* = 1.32). These results underscore the effectiveness of the RAFT polymerization technique in achieving controlled polymer synthesis. Additionally, Fourier-transform infrared (FT-IR) spectroscopy revealed a prominent stretching vibration of the phenyl group at 694 cm^−1^, as shown in Figure 1d. Collectively, these findings confirm the successful synthesis of PTEG_30_-*co*-PPMA_n_ through RAFT polymerization. Furthermore, the thermal decomposition temperatures of all synthesized polymers exceeded 150 °C. However, the incorporation of PPMA blocks reduced the decomposition temperature of the polymer and significantly increased its weight loss percentage. For instance, under identical conditions, PTEG_30_ exhibited a decomposition temperature of 213.5 °C at 5% weight loss with a weight loss percentage of 92.2%, whereas PTEG_30_-*co*-PPMA_120_ showed a reduced decomposition temperature of 199.2 °C and an increased weight loss percentage of 99.2%, as shown in Figure 1e. This indicates that the phenylethanol monomer possesses greater thermal liability.

**Figure 2:**
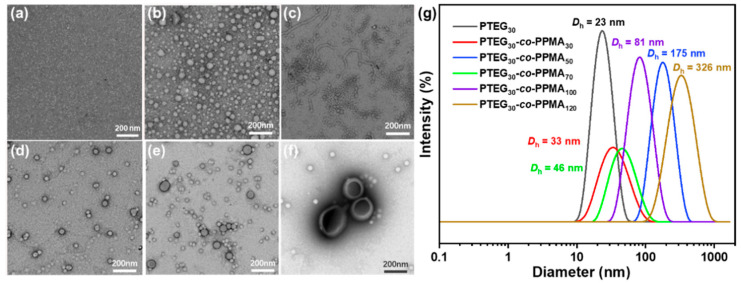
TEM images of (**a**) PTEG_30_, (**b**) PTEG_30_-*co*-PPMA_30_, (**c**) PTEG_30_-*co*-PPMA_50_, (**d**) PTEG_30_-*co*-PPMA_70_, (**e**) PTEG_30_-*co*-PPMA_100_, and (**f**) PTEG_30_-*co*-PPMA_120_. (**g**) DLS data of PTEG_30_ and PTEG_30_-*co*-PPMA_n_.

After polymerization, the solutions became opaque, which implies the formation of nanogels during polymerization [33]. Transmission electron microscopy (TEM) was employed to observe the morphologies of the self-assembly nanogels after being diluted to 0.5 mg/mL. The morphological transition sequence of the assemblies followed the conventional evolutionary pathway of sphere–worm–vesicle–large vesicle (Figure S3) [34,35]. The homopolymer PTEG_30_ failed to form regularly shaped assemblies in the aqueous solution due to the absence of hydrophobic blocks (Figure 2a). Upon introduction of a second PPMA block with a polymerization degree (DP) of 30, micelles with an average diameter of 33 nm were formed through insoluble nucleation block aggregation (Figure 2b). Subsequent extension of the nucleation block induced morphological evolution from individual micelles to micellar aggregates, accompanied by the emergence of worm-like micelles (Figure 2c) [36,37]. Notably, vesicular structures began to appear at a DP of 70, with their diameters progressively increasing upon further chain extension (Figure 2d,e). This progression culminated in the formation of large vesicles at a DP of 120 (Figure 2f). Dynamic light scattering (DLS) measurements revealed a continuous size expansion from 33 nm to 326 nm during this process (Figure 2g and Table S2). An intriguing observation was made at a DP of 50, where the measured hydrodynamic diameter exceeded that at DPs of 70 and 100. This anomaly might be attributed to the presence of elongated worm-like micelles that exhibited enhanced hydrodynamic dimensions [38]. These structural transformations suggest that chain migration and rearrangement driven by the nucleation block played a crucial role in mediating the morphological evolution. The stepwise transitions between different assembly architectures demonstrate the delicate balance between chain mobility and thermodynamic stability during the block extension process [39,40].

### 2.2. Antibacterial Activity of PTEG_30_-co-PPMA_n_

**Figure 3:**
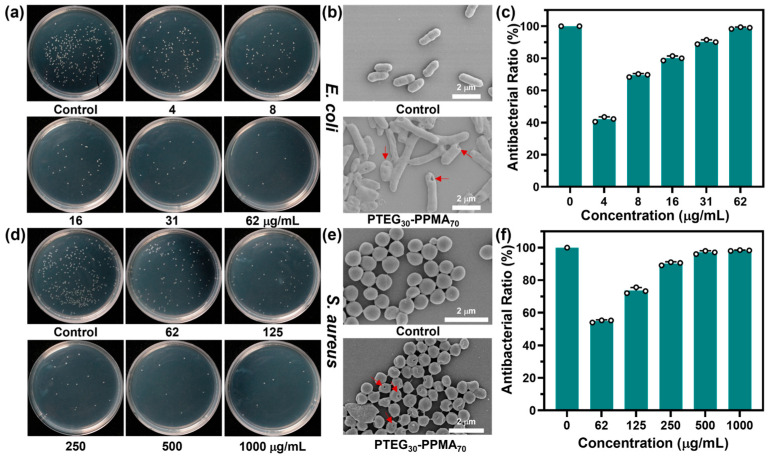
Analysis of antibacterial activity of PTEG_30_-PPMA_n_. Photos of (**a**) *E. coli* and (**d**) *S. aureus* colonies in different treatments. SEM images of PTEG_30_-*co*-PPMA_70_-treated (**b**) *E. coli* and (**e**) *S. aureus*, with PBS-treated samples as a control. The damaged part of the cell is as indicated by the red arrow. Antibacterial ratio of different concentrations of PTEG_30_-*co*-PPMA_70_ against (**c**) *E. coli* and (**f**) *S. aureus* calculated by the number of colonies.

Given that PTEG_30_-*co*-PPMA_n_ contains PPMA components, we first investigated the antibacterial activity of the synthesized nanogels. The antimicrobial efficacy of PTEG_30_-*co*-PPMA_n_ against a broad spectrum of microorganisms (*E. coli* and *S. aureus*) was systematically evaluated by determining the minimum inhibitory concentration (MIC), as summarized in Table S3. Notably, the MIC values for PTEG_30_-*co*-PPMA_30_, PTEG_30_-*co*-PPMA_50_, PTEG_30_-*co*-PPMA_70_, PTEG_30_-*co*-PPMA_100_, and PTEG_30_-*co*-PPMA_120_ against *E. coli* were measured as 128, 128, 62, 62, and 62 μg mL^−1^, respectively, while the corresponding values for *S. aureus* were significantly higher (2000, 2000, 1000, 1000, and 1000 μg mL^−1^). These results demonstrate that PTEG_30_-*co*-PPMA_n_ exhibits superior antibacterial performance against *E. coli* compared with *S. aureus*. In previous work, many reports documented the same findings [41,42,43]. Among the polymers achieving the lowest MIC values, PTEG_30_-*co*-PPMA_70_ was characterized by the lowest degree of polymerization (DP) of the PMA monomer (with an actual polymerization degree of 36), displayed the most pronounced antimicrobial efficacy, and was, consequently, selected for further mechanistic studies. As illustrated in Figure 3a,d, antibacterial assays conducted over 16 h at 37 °C revealed a dose-dependent relationship between the PTEG_30_-*co*-PPMA_70_ concentration and microbial inhibition. When the polymer concentration was increased from 4 to 62 μg mL^−1^, the bactericidal efficiency against *E. coli* escalated dramatically from 42.1% to 98.9% compared with untreated controls (Figure 3c). Concurrently, the antibacterial rate against *S. aureus* reached 52% at 62 μg mL^−1^ (Figure 3f).

We further evaluated the antibacterial performance of this nanogel against multidrug-resistant (MDR) strains, selecting *Pseudomonas aeruginosa* (*P. aeruginosa*) and methicillin-resistant *Staphylococcus aureus* (*MRSA*) for assessment. Based on the antibacterial plate assay results presented in Figure S4, as the polymer concentration increased progressively from 62 µg/mL to 500 µg/mL, the bactericidal efficiency of the nanogel against *MRSA* significantly improved from 15.98% to 76.09%. Conversely, when treating *P. aeruginosa*, the bactericidal efficiency markedly increased from 44.02% to 98.33%. These results demonstrate that the PTEG_30_-*co*-PPMA_70_ nanogel possesses bactericidal capability even against drug-resistant strains. Subsequently, we evaluated the difference in antibacterial efficacy between the nanogel and a commercially available antibiotic, norfloxacin (Nf), using *S. aureus* as the model organism. As shown in Figure S5, the antibacterial rates of both Nf and PTEG_30_-*co*-PPMA_70_ increased significantly with rising concentration. However, the antibacterial performance of PTEG_30_-*co*-PPMA_70_ consistently remained slightly lower than that of Nf. The *t*-test results indicated *p*-values between the antibacterial rates greater than 0.01 but less than 0.05, signifying a statistically significant difference, though not reaching the level of extreme significance. Collectively, these findings demonstrate that the nanogel exhibits superior broad-spectrum antibacterial properties.

To elucidate the antimicrobial mechanism, scanning electron microscopy (SEM) was employed to assess morphological changes and membrane integrity in the treated bacteria. As shown in Figure 3b,e, *E. coli* and *S. aureus* cells exposed to PTEG_30_-*co*-PPMA_70_ exhibited severe structural deformities, including membrane collapse and leakage of cytoplasmic contents. This observation strongly suggests that the polymer disrupts bacterial cell envelope integrity, a critical factor in its bactericidal activity [44]. The combined MIC data, concentration-dependent antibacterial profiles, and SEM-based morphological evidence collectively demonstrate the robust antimicrobial properties of PTEG_30_-*co*-PPMA_70_. To demonstrate the binding interaction between the nanogel and bacteria, zeta potential measurements were performed, as shown in Figure S6. The results indicated that PTEG_30_-*co*-PPMA_70_ itself exhibited a zeta potential of −33.1 mV, attributed to the absence of positively charged groups and the presence of carboxyl groups from the chain-transfer agent (CTA). The zeta potentials of *S. aureus* and *E. coli* were measured as −31 mV and −27 mV, respectively. Upon increasing the PTEG_30_-*co*-PPMA_70_ concentration from 0 to 0.8 mg mL^−1^, the zeta potential of *S. aureus* slightly declined from −31 mV to −35 mV. In contrast, a more pronounced change was observed for *E. coli*, where its zeta potential decreased from −27 mV to −37 mV. These findings suggest stronger binding of PTEG_30_-*co*-PPMA_70_ to the Gram-negative *E. coli*. This enhanced binding correlates with the observed increase in efficacy against Gram-negative *E. coli*, which is likely attributable to structural differences in the bacterial cell walls. This necessitates further investigation into the species-specific nature of these interactions [45,46].

To evaluate the biocompatibility of the nanogel, PTEG_30_-*co*-PPMA_70_ was systematically tested through hemolysis assays and MTT assays. The experimental results revealed that when the concentration of P3 ranged from 62 to 1000 μg mL^−1^, its hemolysis ratio remained below 3% (Figure S7a). This indicates that PTEG_30_-*co*-PPMA_70_ causes minimal disruption to erythrocyte membranes and exhibits excellent hemocompatibility. Furthermore, no significant decline in the viability of HEK293T cells was observed with an increasing PTEG_30_-*co*-PPMA_70_ concentration. Even at concentrations as high as 1 mg mL^−1^, cell viability remained above 90% (Figure S7b). These findings position PTEG_30_-*co*-PPMA_70_ as a promising candidate for antimicrobial applications requiring targeted pathogen suppression.

## 3. Conclusions

In summary, a series of amphiphilic block copolymers (PTEG_30_-*co*-PPMA_n_) with tunable molecular weights and well-defined architectures was successfully synthesized via RAFT polymerization. Comprehensive characterization using ^1^H NMR, GPC, and FT-IR confirmed the controlled synthesis and structural integrity of the copolymers, which exhibited excellent thermal stability with decomposition temperatures above 150 °C. The self-assembly behavior of these polymers in aqueous solutions revealed a morphological evolution from spherical micelles to worm-like nanogels and, eventually, to vesicles as the hydrophobic PPMA block length increased. This structural transition, driven by chain mobility and thermodynamic balance, was corroborated by TEM and DLS analyses, highlighting the critical role of hydrophobic block extension in modulating the self-assembly nanogel architecture. Antibacterial evaluations demonstrated that PTEG_30_-*co*-PPMA_n_ exhibited significant antimicrobial activity, particularly against Gram-negative *E. coli*, with MIC values as low as 62 μg mL^−1^. The superior performance of PTEG_30_-co-PPMA_70_, attributed to its optimal hydrophobic–hydrophilic balance, underscores the importance of molecular design in enhancing antimicrobial efficacy. SEM imaging revealed that the bactericidal mechanism involves disruption of bacterial membrane integrity, leading to cytoplasmic leakage and cell death. The observed disparity in activity between Gram-negative and Gram-positive species suggests potential selectivity influenced by differences in cell wall structure. Furthermore, while its antibacterial activity is slightly lower than that of the conventional antibiotic norfloxacin, no statistically significant difference was observed. Additionally, the nanogel exhibited excellent biocompatibility. This study not only advances the understanding of structure–property relationships in amphiphilic copolymers but also provides a versatile platform for designing functional nanogels with tailored antimicrobial properties. Future work should focus on optimizing polymer architectures for targeted applications and exploring synergistic effects with conventional antibiotics to broaden their therapeutic potential.

## 4. Materials and Methods

### 4.1. Materials

Tetraethylene glycol (99%), phenethyl alcohol (PMA, Analytical Reagent, AR), triethylamine (TEA, AR), and iodine (I_2_ 99%) were obtained from Aladdin (Shanghai, China). Methacryloyl chloride (MAC, AR) and methacrylic anhydride (MAC, AR) were obtained from Rhawn (Shanghai, China). 4-cyano-4-(thiobenzoylthio) pentanoic acid (CTA, 97%) and 2,2′-azobis(2-methylpropionitrile) (AIBN, 99%) were purchased from J&K Scientific (Beijing, China).

### 4.2. Instruments

Proton nuclear magnetic resonance (^1^H NMR) spectra were recorded using an Agilent DD2 600 MHz from Bruker BioSpin International (MA, USA), using CDCl_3_ as the solvent. The solvent peaks of deuterated reagents (CDCl_3_: 7.26 ppm) were used as a reference for chemical shifts. Fourier-transform infrared (FT-IR) spectra were measured in the range of 4000−400 cm^−1^ by a Nicolei 6670 FT-IR spectrometer (MA, USA). Thermal stability was characterized via a METZSCH TG 209 F3 tarsus thermogravimetric analyzer (TGA) (Bavaria, Germany). The gel permeation chromatography (GPC) measurements were conducted by employing a PLgel 5 mm (CA, USA) mixed C column and a guard column. HPLC-grade DMF containing 5 mM NH_4_BF_4_ was used as the mobile phase, and the analysis was performed at a temperature of 50 °C with a flow rate of 1.0 mL min^−1^. Prior to the analysis, the GPC system was calibrated by using PMMA as the reference standard. The obtained data were then processed and analyzed by using the Agilent 1260 GPC software (CA, USA). The dynamic light scanning (DLS) data were recorded by Delsa Max Pro from Beckman Coulter (CA, USA). The morphology of assembly was observed by transmission electron microscopy (TEM, HT-7700 microscope, Hitachi, Tokyo, Japan). Scanning electron microscopy (SEM) images were collected with an S-4800 microscope (Hitachi, Tokyo, Japan).

### 4.3. Synthesis of TEG

Tetraethylene glycol (5 g; 25.74 mmol) and TEA (2.61 g; 25.74 mmol) were dissolved in 30 mL of DCM. Methacryloyl chloride (2.69 g; 25.74 mmol) was dissolved in 20 mL of DCM and then added dropwise into the reaction in a N_2_ atmosphere. Then, the mixture was reacted at 20 °C for 18 h. The crude product was purified by column chromatography to obtain TEG.

### 4.4. Synthesis of PMA

Phenethyl alcohol (5 g; 43.38 mmol) and methacrylic anhydride (3.35 g; 21.7 mmol) were dissolved in 100 mL of DCM. I_2_ (1.5 g; 5.91 mmol) was dissolved in 5 mL of DCM and then added dropwise into the reaction in a N_2_ atmosphere. Then, the mixture was refluxed at 60 °C for 24 h. The crude product was purified by column chromatography to obtain PMA. The chemical structure of PMA was confirmed through nuclear magnetic resonance (^1^H NMR) spectroscopy, with the results presented in Figure S2.

### 4.5. Synthesis of Macro-CTA PTEG_30_

TEG (1 g; 3.814 mmol), CTA (35 mg; 0.125 mmol), and AIBN (1.3 mg; 7.9 mmol) were dissolved in 5 mL of THF. After three cycles of freeze–thaw, the mixed solution was reacted at 70 °C in a N_2_ atmosphere for 24 h. Subsequently, it was dialyzed with deionized water (MWCO = 3.5 kDa) for 48 h. After liquid freeze drying, the macro-CTA PTEG_30_ was obtained [47].

### 4.6. Representative Synthesis of PTEG_30_-co-PPMA_100_

PTEG_30_ (60 mg; 5.61 μmol), PMA (107 mg; 0.561 mmol), and AIBN (0.24 mg; 1.462 mmol) were dissolved in 666 μL of MeOH. After three cycles of freeze–thaw, the mixed solution was reacted at 70 °C in a N_2_ environment for 24 h. Subsequently, it was dialyzed with deionized water (MWCO = 3.5 kDa) for 48 h to obtain PTEG_30_-*co*-PPMA_100_. PTEG_30_-*co*-PPMA_30_, PTEG_30_-*co*-PPMA_50_, PTEG_30_-*co*-PPMA_70_, and PTEG_30_-*co*-PPMA_120_ were synthesized using a similar method, with the only variation being the amounts of PMA added to the solutions, which were 32.1 mg, 53.5 mg, 74.9 mg, and 128.4 mg, respectively [48].

### 4.7. MIC Measurements

The MIC of PTEG_30_-*co*-PPMA_n_ against bacteria was determined by the broth microdilution method. PTEG_30_-*co*-PPMA_n_ was gradient-diluted with LB in 96-well plates to obtain 100 μL in different concentrations. The bacteria after 12 h of incubation were washed with PBS and then resuspended in LB to a final concentration of 1.0 × 10^5^ CFU/mL. An amount of 100 μL of bacteria was added to 96-well plates and incubated with different concentrations of the sample at 37 °C for 20 h. The optical density (OD) at 600 nm was always monitored. The MIC was defined as the minimum sample concentration at which bacteria did not grow appreciably. Broth without bacterial inoculation and broth containing microbial cells alone were used as a negative control and a positive control, respectively. All groups were repeated three times [49].

### 4.8. In Vitro Antibacterial Test

To assess antimicrobial activity, *Escherichia coli* (*E. coli*, ATCC 25922) and *Staphylococcus aureus* (*S. aureus*, ATCC 33591) were employed as the test organisms. Following a 12 h cultivation period, the bacterial cultures were subjected to PBS washing and subsequently adjusted to a density of 1.0 × 10^5^ CFU/mL in PBS buffer. For the antimicrobial assay, 100 μL of bacterial suspension was combined with 900 μL of various sample concentrations and maintained at 37 °C for 30 min. Subsequently, 50 μL aliquots from each mixture were plated onto LB agar medium and incubated at 37 °C for 16 h. Colony enumeration was performed to determine viable bacterial counts. Parallel experiments using PBS-treated bacteria served as negative controls. Antimicrobial efficacy was quantified using the following formula:

[(CFU_control_ − CFU_sample_)/CFU_control_] × 100% (1)

### 4.9. Bacterial Morphological Observation

Following a 12 h incubation period, the bacterial cultures were washed with PBS and adjusted to an OD_600_ of 0.1 using PBS. The bacterial suspensions were then exposed to 500 µg/mL of PTEG_30_-*co*-PPMA_100_, respectively, and incubated at 37 °C for 3 h under constant shaking at 220 rpm. After treatment, the bacterial suspensions were carefully deposited onto clean silicon wafers and allowed to adhere for 1 h. Excess liquid was removed using filter paper, and the samples were fixed with a 5% glutaraldehyde solution at 4 °C for 12 h. Subsequently, the adhered bacteria were subjected to a series of ethanol dehydration steps (30%, 50%, 70%, 80%, 90%, and 100%). The samples were air-dried at room temperature for 6 h, followed by gold coating for 30 s, and finally examined under a scanning electron microscope [50,51,52,53,54,55,56].

### 4.10. Zeta Potential Measurement

To prove the binding interaction between the nanogel and bacteria, *Escherichia coli* (*E. coli*, ATCC 25922) and *Staphylococcus aureus* (*S. aureus*, ATCC 33591) were selected as the test strains. After culturing at 37 °C for 12 h, the bacterial cultures were washed with PBS, and their optical density (OD_600_) was adjusted to 0.1 using PBS, followed by a 10-fold dilution with PBS buffer. An amount of 500 μL of the bacterial suspension was mixed with 500 μL of PBS buffer and samples of different concentrations, respectively, so that the final concentrations of PTEG_30_-*co*-PPMA_70_ were 0 µg/mL, 100 µg/mL, 200 µg/mL, 400 µg/mL, and 800 µg/mL, and then incubated at 37 °C for 30 min. Free PTEG_30_-*co*-PPMA_70_ nanogel without bacteria was used as the control. All groups were measured three times.

### 4.11. Hemolysis Assay

Sheep red blood cells (RBCs) at a 4% concentration were prepared by centrifugation (5000 rpm; 5 min) and washed three times with phosphate-buffered saline (PBS). Following washing, the RBCs were reconstituted to their original volume in PBS. Aliquots (500 µL) of PTEG_30_-*co*-PPMA_70_ solutions at varying concentrations were mixed with an equal volume (500 µL) of the RBC suspension to achieve final PTEG_30_-*co*-PPMA_70_ concentrations of 62, 125, 250, 500, and 1000 µg mL^−1^. After incubation at 37 °C for 1 h, the mixtures were centrifuged again (5000 rpm; 5 min). The supernatant (100 µL) from each sample was then transferred to a 96-well plate for absorbance measurement at 560 nm (OD_560_). Negative (0% hemolysis) and positive (100% hemolysis) controls consisted of RBCs treated with PBS and 1% Triton X-100, respectively [38]. The percentage hemolysis was calculated using the following formula:

hemolysis (%) = [(OD_560_ sample − OD_560_ negative control)/(OD_560_ positive control − OD_560_ negative control)] × 100% (2)

All treatments were performed in triplicate.

### 4.12. MTT Test

HEK293T cells were seeded in 96-well plates and incubated with PTEG_30_-*co*-PPMA_70_ at a fixed concentration for 24 h. Subsequently, MTT solution (5 mg/mL) was added and incubated for 4–6 h. The optical density (OD) of each well was measured at 540 nm using a Multiskan MK3 microplate reader (Thermo Scientific, Waltham, MA, USA).

## Data Availability

The data presented in this study are openly available in this article.

## Supplementary Materials

The following supporting information can be downloaded at https://www.mdpi.com/article/10.3390/gels11090690/s1. Table S1. Molecular weights of polymers. Table S2. DLS data of PTEG_30_-*co*-PPMA_n_. Table S3. Minimal inhibitory concentrations (MICs) of PTEG_30_-*co*-PPMA_n_ against *E. coli* and *S. aureus*. Figure S1. ^1^H NMR spectra of PTEG_30_ polymer in CDCl_3_. Figure S2. ^1^H NMR spectra of PMA monomer in CDCl_3_. Figure S3. TEM images of (a) PTEG_30_, (b) PTEG_30_-*co*-PPMA_30_, (c) PTEG_30_-*co*-PPMA_50_, (d) PTEG_30_-*co*-PPMA_70_, (e) PTEG_30_-*co*-PPMA_100_, and (f) PTEG_30_-*co*-PPMA_120_. Figure S4. Analysis of antibacterial activity of PTEG_30_-*co*-PPMA_70_. Figure S5. Analysis of antibacterial activities of antibiotic norfloxacin and PTEG_30_-*co*-PPMA_70_. Figure S6. Analysis of zeta potential changes of *S. aureus* and *E. coli* after treatment with different concentrations of PTEG_30_-*co*-PPMA_70_, respectively. Figure S7. Hemolysis assessment and cytotoxicity analysis of PTEG_30_-*co*-PPMA_70_.
