# Supplementary material for: Phenylethyl Alcohol-Based Polymeric Nanogels Obtained Through Polymerization-Induced Self-Assembly Toward Achieving Broad-Spectrum Antibacterial Activity
Source: Gels. 2025 Sep 1;11(9):690. doi: 10.3390/gels11090690 (PMC12469702; doi:10.3390/gels11090690)
Supplement: Supplementary file 1 [file gels-11-00690-s001.zip › gels-3766553-supplementary.pdf]

Supplementary information

of

Phenylethyl Alcohol-based Polymeric Nanogels through Polymerization-Induced Self-Assembly toward Broad Spectrum Antibacterial Activity

[a] Department of Material Science and Engineering, School of Material and Chemistry, Anhui Agricultural University, Hefei, Anhui 230036, China

[b] Key Laboratory of Jiangxi Province for Functional Biology and Pollution Control in Red Soil Regions, Jinggangshan University, Ji' an, Jiangxi 343009, China

Corresponding Authors

Qiaoran Li – *Hefei, Anhui 230036, China*; Email: liqiaoran@stu.ahau.edu.cn

Feihu Bi – *Ji' an, Jiangxi 343009, China*; Email: bifeihu@jgsu.edu.cn

Content

Table S1. Molecular weight of polymers....3

Table S2. Hydrodynamic diameter obtained from DLS measurement....3

Table S3. Minimal inhibitory concentrations (MIC) of $PTEG_{30}$-*co*-$PPMA_n$ against *E. coli* and *S. aureus.*....3

Figure S1. ^{1}H NMR spectra of polymer $PTEG_{30}$ in $CDCl_3$....4

Figure S2. ^{1}H NMR spectra of monomer PMA in $CDCl_3$....4

Figure S3. TEM images of (a) $PTEG_{30}$, (b) $PTEG_{30}$-*co*-$PPMA_{30}$, (c) $PTEG_{30}$-*co*-$PPMA_{50}$, (d) $PTEG_{30}$-*co*-$PPMA_{70}$, (e) $PTEG_{30}$-*co*-$PPMA_{100}$, (f) $PTEG_{30}$-*co*-$PPMA_{120}$....5

Figure S4. Analysis of antibacterial activity of $PTEG_{30}$-*co*-$PPMA_{70}$....5

Figure S5. Analysis of the antibacterial activities of the antibiotic norfloxacin and $PTEG_{30}$-*co*-$PPMA_{70}$....5

Figure S6. Analysis of the zeta potential changes of *S. aureus* and *E. coli* after treatment with different concentrations of $PTEG_{30}$-*co*-$PPMA_{70}$, respectively....6

Figure S7. The hemolysis assessment and cytotoxicity analysis of $PTEG_{30}$-*co*-$PPMA_{70}$....6

Table S1. Molecular weight of polymers.

Polymer	DP, entry[a]	DP,NMR[b]	Conv. (%)[c]	$M_{n,\ GPC}$[d]	$M_{w,\ NMR}$[e]	Đ
$PTEG_{30}$	30	28	93.3	10600	7600	1.19
$PTEG_{30}$-*co*-$PPMA_{30}$	30	20	66.7	12000	11400	1.24
$PTEG_{30}$-*co*- $PPMA_{50}$	50	28	52.8	13400	12900	1.30
$PTEG_{30}$-*co*- $PPMA_{70}$	70	36	51.4	14600	14400	1.26
$PTEG_{30}$-*co*- $PPMA_{100}$	100	60	60.0	15200	19000	1.26
$PTEG_{30}$-*co*- $PPMA_{120}$	120	73	60.8	16200	21400	1.32

[a] DP, entry represented the theoretical entry of PMA.

[b] Determined by means of ^{1}H NMR analysis.

[c] The monomer conversion determined by ^{1}H NMR analysis.

[d,e] Mn (g/ mol) (number-average molecular weight) and Mw (g/ mol) (weight-average molecular weight) were determined by GPC analysis and ^{1}H NMR analysis.

Table S2. Hydrodynamic diameter obtained from DLS measurement.

Polymer	D_h (nm)
$PTEG_{30}$	23 ± 4.5
$PTEG_{30}$-*co*-$PPMA_{30}$	33 ± 3.7
$PTEG_{30}$-*co*- $PPMA_{50}$	175 ± 33.1
$PTEG_{30}$-*co*- $PPMA_{70}$	46 ± 11.1
$PTEG_{30}$-*co*- $PPMA_{100}$	81 ± 15.1
$PTEG_{30}$-*co*- $PPMA_{120}$	326 ± 43.1

Table S3. Minimal inhibitory concentrations (MIC) of $PTEG_{30}$-*co*-$PPMA_n$ against *E. coli* and *S. aureus.*

**Sample**	***E.coli***	***S.aureus***
$PTEG_{30}$-*co*-$PPMA_{30}$	128	2000
$PTEG_{30}$-*co*-$PPMA_{50}$	128	2000
$PTEG_{30}$-*co*-$PPMA_{70}$	62	1000
$PTEG_{30}$-*co*-$PPMA_{100}$	62	1000
$PTEG_{30}$-*co*-$PPMA_{120}$	62	1000

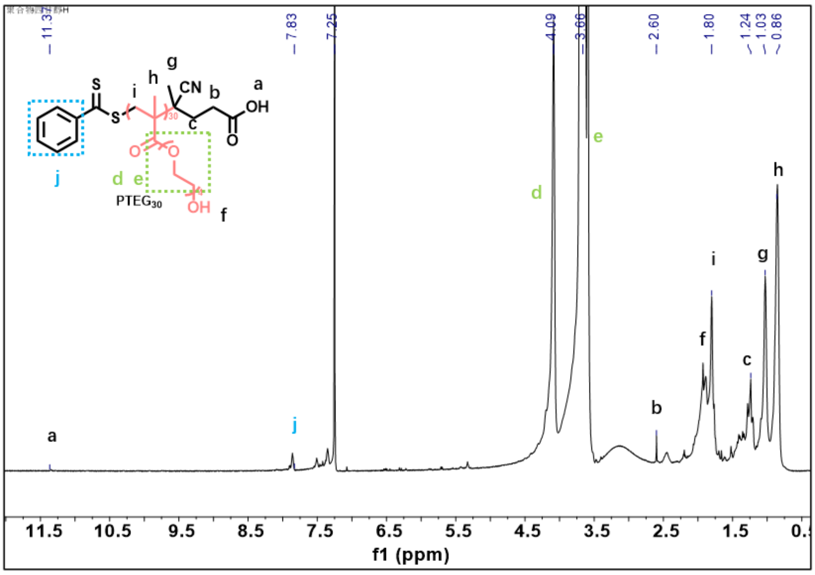

Figure S1. ^{1}H NMR spectra of polymer $PTEG_{30}$ in $CDCl_3$.

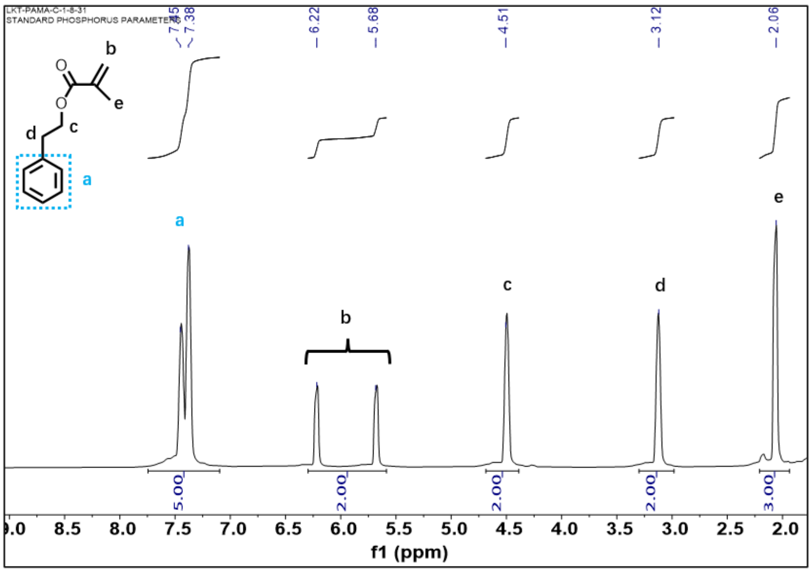

Figure S2. ^{1}H NMR spectra of monomer PMA in $CDCl_3$.

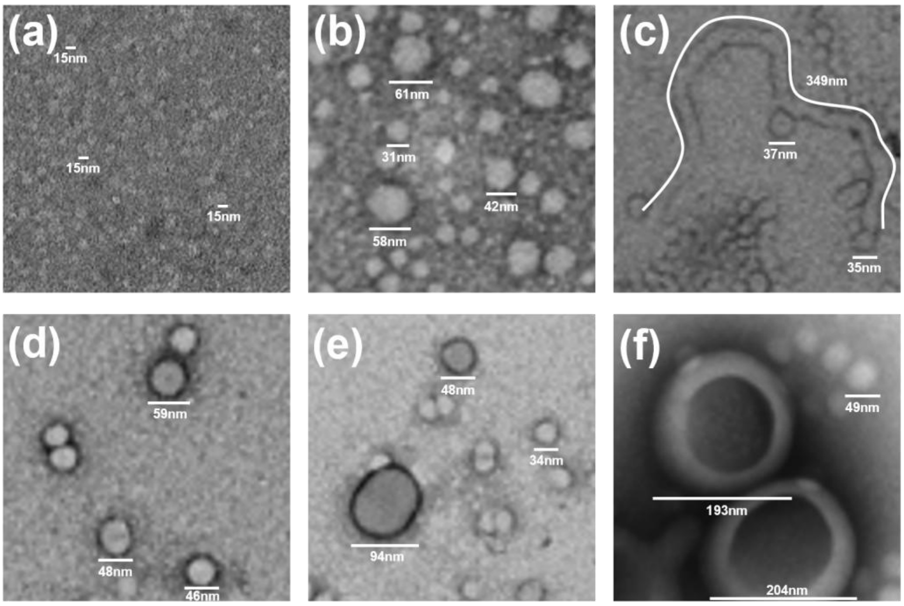

Figure S3. TEM images of (a) $PTEG_{30}$, (b) $PTEG_{30}$-*co*-$PPMA_{30}$, (c) $PTEG_{30}$-*co*-$PPMA_{50}$, (d) $PTEG_{30}$-*co*-$PPMA_{70}$, (e) $PTEG_{30}$-*co*-$PPMA_{100}$, (f) $PTEG_{30}$-*co*-$PPMA_{120}$.

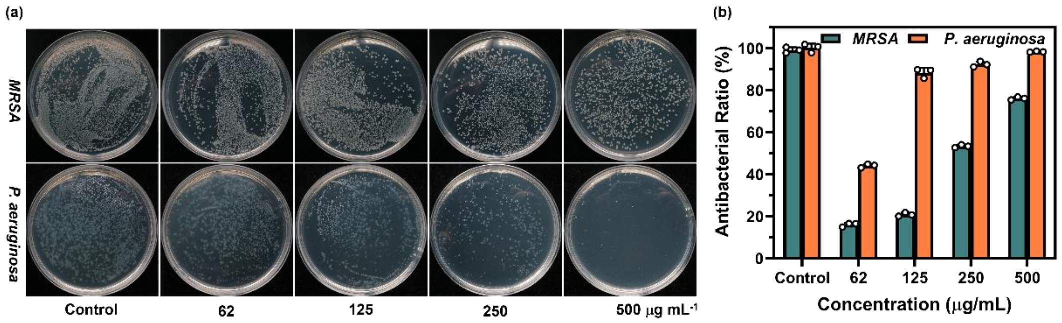

Figure S4. Analysis of antibacterial activity of $PTEG_{30}$-*co*-$PPMA_{70}$.

(a) Photographs of *Pseudomonas aeruginosa* and methicillin-resistant *Staphylococcus aureus* colonies treated with different concentrations of $PTEG_{30}$-*co*-$PPMA_{70}$. (b) Calculation of the antibacterial rates of different concentrations of $PTEG_{30}$-*co*-$PPMA_{70}$ against *Pseudomonas aeruginosa* and methicillin-resistant *Staphylococcus aureus* based on the number of colonies.

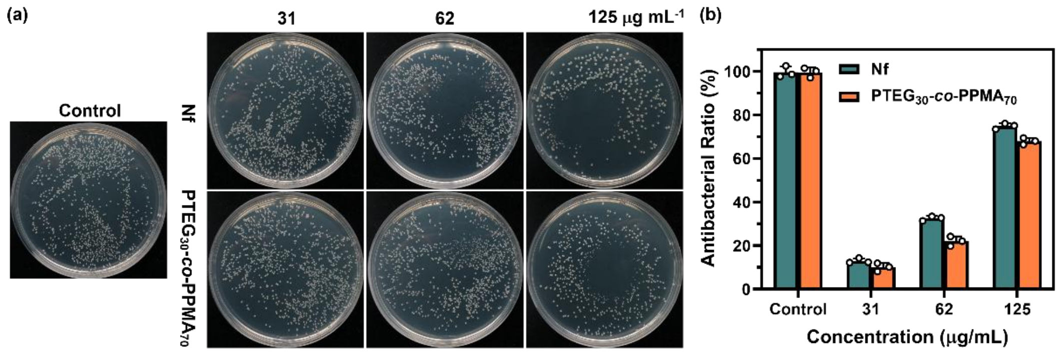

Figure S5. Analysis of the antibacterial activities of the antibiotic norfloxacin and $PTEG_{30}$-*co*-$PPMA_{70}$.

(a) Photos of *MRSA* colonies treated with different concentrations of norfloxacin and PTEG$_{30}$-*co*-PPMA$_{70}$. (b) Calculation of the antibacterial rates of different concentrations of norfloxacin and PTEG$_{30}$-*co*-PPMA$_{70}$ against *MRSA* based on the number of colonies.

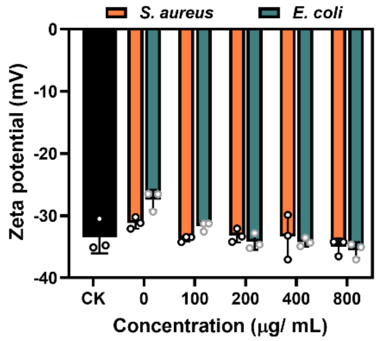

Figure S6. Analysis of the zeta potential changes of *S. aureus* and *E. coli* after treatment with different concentrations of PTEG$_{30}$-*co*-PPMA$_{70}$, respectively.

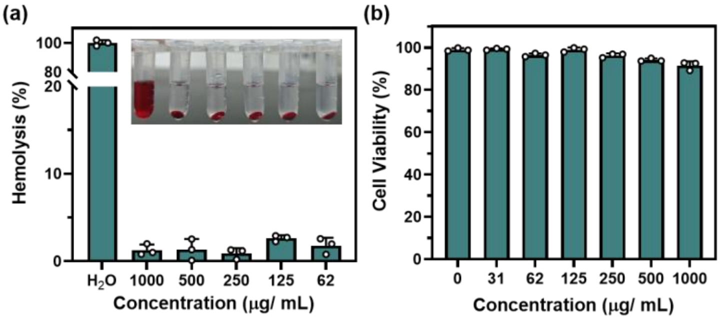

Figure S7. The hemolysis assessment and cytotoxicity analysis of PTEG$_{30}$-*co*-PPMA$_{70}$.

(a) Hemolytic activity of PTEG$_{30}$-*co*-PPMA$_{70}$ was evaluated by incubating the copolymer with 4% sheep erythrocytes. Digital photograph of centrifuged blood cells after 1 h incubation with PTEG$_{30}$-*co*-PPMA$_{70}$ at concentrations ranging from 0 to 1000 μg mL^{-1}. (b) Viability of HEK293T cells was measured following treatment with PTEG$_{30}$-*co*-PPMA$_{70}$ across concentrations of 0-250 μg mL^{-1}.
